# Machine-learning prediction of a novel diagnostic model using mitochondria-related genes for patients with bladder cancer

**DOI:** 10.1038/s41598-024-60068-9

**Published:** 2024-04-23

**Authors:** Jian Li, Zhiyong Wang, Tianen Wang

**Affiliations:** Department of Urology, The First Affiliated Hospital of Zhengzhou University, Zhengzhou, China

**Keywords:** Mitochondria, Bladder cancer, Diagnostic model, Machine Learning, Immune infiltration, GEO datasets, Cancer, Genetics, Immunology

## Abstract

Bladder cancer (BC) is the ninth most-common cancer worldwide and it is associated with high morbidity and mortality. Mitochondrial Dysfunction is involved in the progression of BC. This study aimed to developed a novel diagnostic model based on mitochondria-related genes (MRGs) for BC patients using Machine Learning. In this study, we analyzed GSE13507 datasets and identified 752 DE-MRGs in BC specimens. Functional enrichment analysis uncovered the significant roles of 752 DE-MRGs in key processes such as cellular and organ development, as well as gene regulation. The analysis revealed the crucial functions of these genes in transcriptional regulation and protein-DNA interactions. Then, we performed LASSO and SVM-RFE, and identified four critical diagnostic genes including GLRX2, NMT1, OXSM and TRAF3IP3. Based on the above four genes, we developed a novel diagnostic model whose diagnostic value was confirmed in GSE13507, GSE3167 and GSE37816 datasets. Moreover, we reported the expressing pattern of GLRX2, NMT1, OXSM and TRAF3IP3 in BC samples. Immune cell infiltration analysis revealed that the four genes were associated with several immune cells. Finally, we performed RT-PCR and confirmed NMT1 was highly expressed in BC cells. Functional experiments revealed that knockdown of NMT1 suppressed the proliferation of BC cells. Overall, we have formulated a diagnostic potential that offered a comprehensive framework for delving into the underlying mechanisms of BC. Before proceeding with clinical implementation, it is essential to undertake further investigative efforts to validate its diagnostic effectiveness in BC patients.

## Introduction

Bladder cancer (BC) is the sixth most common cancer in men that is characterized by high risks of recurrence and mortality^1^. BC is more prevalent in males than in females, with males having a risk approximately 3 to 4 times higher than females. This discrepancy could be attributed to the higher smoking rates among males and their increased exposure to occupational chemicals^2^. The occurrence of BC is primarily observed in the middle-aged to elderly population, with an average age around 60 years. However, there are also cases of BC among younger individuals, although their proportion is relatively small. The incidence of BC varies globally. Developed countries generally experience higher rates, possibly owing to elevated smoking rates, levels of industrialization, and dietary habits. For instance, BC incidence is higher in countries like the United States, Canada, and European nations^3,4^. In contrast, the incidence might be relatively lower in developing countries, but with the increase in smoking rates and changes in lifestyle, these disparities could gradually diminish over time. Despite much research and significant progress over the past few decades, the 5-year survival rate still falls below expectations. Therefore, the discovery of new biomarkers for prognosis and individualized therapy in BC holds considerable significance.

Mitochondria are a crucial organelle within cells responsible for energy production and regulation of cellular metabolism^5^. In addition to these fundamental functions, mitochondria also play significant roles in processes such as cell apoptosis, cell signaling, and calcium ion balance^6^. The role of mitochondria in maintaining cellular function and health cannot be overlooked. Tumor cells exhibit differences in energy metabolism compared to normal cells. While normal cells primarily generate the majority of their energy through oxidative phosphorylation, tumor cells tend to favor glycolysis (an anaerobic metabolic pathway) for energy production, even in the presence of sufficient oxygen^7,8^. This phenomenon is known as the "Warburg effect," wherein alterations in mitochondrial function may play a pivotal role in the metabolic pathways of tumor cells^9,10^. Certain tumors may have impaired mitochondrial function, which could be related to their growth and invasive abilities. Abnormal mitochondrial function might disrupt cell death pathways, inhibiting the normal progression of apoptosis, and consequently contribute to the survival and spread of tumor cells. Oxidative stress generated by mitochondria could potentially lead to damage of cellular DNA and other biomolecules, thereby increasing the risk of tumor development^11^. Mitochondrial damage could result in mutations of cellular genetic material, possibly being a contributing factor in tumor formation^12,13^. In the context of BC, previous researches have indicated that abnormalities in mitochondrial function, such as changes in mitochondrial DNA, alterations in mitochondrial membrane potential, and shifts in mitochondrial dynamics, may contribute to tumorigenesis, tumor growth, and resistance to therapy^14,15^. In addition, some studies suggest that the functionality of mitochondria may be compromised in the tumor tissue of BC patients. The progress could involve mutations in mitochondrial DNA, changes in mitochondrial membrane potential, and an increase in oxidative stress. These abnormalities could lead to disruptions in energy metabolism, impact cell apoptosis pathways, and consequently contribute to the survival and proliferation of cancer cells^16,17^. However, the potential function of mitochondria-related genes (MRGs) remained largely unclear. Given the important roles of MRGs in BC progression, it is important to screen novel biomarkers based on MRGs for BC patients.

Machine Learning is a subfield of Artificial Intelligence (AI) that aims to enable computer systems to automatically learn and improve from data, in order to perform specific tasks without explicit programming^18^. The goal of machine learning is to empower computer systems to extract patterns, trends, and knowledge from data, and make predictions, classifications, recognitions, or decisions based on new data. Machine learning combined with transcriptome sequencing plays a crucial role in the screening of diagnostic biomarkers for tumors^19,20^. Through transcriptome sequencing, we can obtain rich information about the gene expression of tumor cells. Leveraging the powerful capabilities of machine learning, key features can be extracted from vast datasets to identify gene expression patterns associated with tumors. These patterns can be integrated into classification and prediction models, aiding in the diagnosis of different types of tumors, predicting tumor progression, and even tailoring personalized treatment plans based on individual transcriptome data^21,22^. Additionally, machine learning contributes to uncovering the molecular mechanisms behind tumor development, providing scientists and doctors with deeper insights. By integrating multi-omics data, such as proteomics and metabolomics, the accuracy and reliability of diagnostic biomarkers can be further enhanced. Overall, the integration of machine learning and transcriptome sequencing brought new hope to the field of tumor diagnosis, providing robust support for precision medicine and personalized treatment. In this study, we aimed to develop a novel diagnostic model based on MRGs for BC patients using Machine Learning and Transcriptome sequencing.

## Materials and methods

### Cell lines and culture

The Chinese Academy of Sciences' Cell Bank in Shanghai supplied a regular human bladder uroepithelium cell line, SV-HUC-1, and four distinct BC cell lines, namely 5637, J82, T24, and UMUC3. SV-HUC-1, T24, and UMUC3 were propagated in DMEM from Gibco, located in Carlsbad, CA. For the growth of 5637 and J82 lines, the medium chosen was RPMI-1640, also from Gibco. Each culture medium was supplemented with 1% fetal bovine serum (FBS) and 1% penicillin/streptomycin. These cells thrived in an environment maintained at 37 °C, under 5% CO_2_, with a humid atmosphere.

#### N-myristoyltransferase 1(NMT1) siRNA and transfection

Post a 24-h incubation period at 37 degrees Celsius, 1 × 10^6^ cells of J82 and UMUC3 were distributed into 6-well plates by Corning, Inc. (Corning, NY, USA). A 4 µg dose of specific siRNA aimed at NMT1 or its control counterpart was introduced to 250 µl of DMEM devoid of serum and stirred thoroughly. Concurrently, 10 µl of Lipofectamine 2000, sourced from Thermo Fisher Scientific (Waltham, MA, USA), was integrated into a separate 250 µl serum-free DMEM, given a gentle stir and set aside at ambient temperature for a brief 5-min span. Subsequently, a thorough amalgamation of both siRNA and Lipofectamine 2000 was achieved, followed by a 20-min stabilization period at ambient conditions. This concoction was then introduced into the 6-well plates, softly agitated for uniformity, and subjected to a 37 °C environment. Post 6 h from transfection initiation, the cell culture's medium was substituted, enriching every well with 2 ml of fresh medium. Post-transfection at the 48-h mark, cells were collated for ensuing procedures, with unaltered cells marking the standard reference. A confirmation of the transfection's success was ascertained through RT-PCR.

### Real-time PCR

RNA extraction was undertaken using the TRIzol reagent procedures from CWBio (Suzhou, Jiangsu, China). The progress was followed by cDNA synthesis, facilitated by kits from Takara (Dalian, China). Subsequent NMT1 qPCR analysis utilized SYBR Green qPCR kits provided by Haihong (Ningbo, Zhejiang, China), with specific conditions set at 95 °C for 5 min and 39 cycles encompassing 95 °C for 20 s and 72 °C for 3 min. NMT1's fold changes were determined employing the 2^−ΔΔCt^ approach. Notably, GAPDH functioned as the reference control in the NMT1 detection. The primers were listed as follows: GAPDH: forward 5’-GGAGCGAGATCCCTCCAAAAT-3’ and reverse 5’- GGCTGTTGTCATACTTCTCATGG-3’; NMT1: forward 5’- GGTCAGGGACCTGCCAAAAC-3’ and reverse 5’- CATGGGTGTTCACCACTTCG-3’.

#### Cell counting kit-8 (CCK-8) assay

We initiated by seeding J82 and UMUC3 cells into 96-well plates, maintaining a density of 2 × 10^3^ cells for each well. For a span of 24 h, these cells underwent CO_2_ incubation to ensure thorough adherence. Subsequently, a 10 μL CCK-8 solution (Dojindo Co., Tokyo, Japan) was added to each well, followed by a 2.5 h incubation at 37 °C. Using a microplate reader, the optical density (OD) was assessed at a 450 nm wavelength. Post the OD measurement, the spent medium in each well was replaced. OD recording process was consistent, taking place every 24 h over a week. From these OD readings, a growth curve was derived. Each experimental set was executed thrice for consistency.

### Data collection

Gene Expression Omnibus (GEO) database, gathers a comprehensive set of gene expression recordings derived from an array of biological sources(https://www.ncbi.nlm.nih.gov/gds). This spans from RNA-seq to microarray tests. It's a reservoir of information from multifaceted biological investigations, which capture expression signatures from a wide spectrum of tissues, cells, and various biological conditions. The database serves as a platform for scientists to explore, query, and retrieve these datasets, facilitating diverse bioinformatics exploration and scholarly inquiries. For our study, RNA-sequence data of both BC and standard samples were extracted, specifically from the datasets GSE13507^23^, GSE3167^24^ and GSE37816^25^. Furthermore, we screened MRGs with a count of 1513 from MSigDB (https://www.gsea-msigdb.org/gsea/msigdb).

#### Differential expression analysis

From the GSE13507 database, we sourced the expression profiles for 1513 MRGs present in both standard and BC specimens. Then, using the R platform, we employed the student's t-test to identify any MRGs exhibiting differential expression between the two sample sets. A p-value less than 0.05 was deemed indicative of significance.

#### Functional enrichment analysis of DE-MRGs

The Gene Ontology (GO) system offers a structured, standardized nomenclature to delineate gene functions and associated biological pathways. This framework enables insights into gene interactions and their roles within organisms. Similarly, Kyoto Encyclopedia of Genes and Genomes (KEGG) serves as an exhaustive bioinformatics resource, shedding light on topics spanning genomics, biochemistry, and systems biology^26^. Such insights from KEGG include details on genes, proteins, metabolic pathways, and even pharmaceutical compounds. To unravel the potential functionalities and pathways of pivotal genes, both GO and KEGG enrichment analyses were undertaken using clusterProfiler. Recognized in the bioinformatics field, clusterProfiler is a go-to R package crafted for diving into high-throughput biological datasets to uncover functional and pathway relevance. It propels the understanding of biological significance tied to specific gene clusters, decoding the roles and connections among various genes or proteins. For a clear representation of the analytical findings, clusterProfiler's visualization tool was employed. A significance threshold was established at *P* < 0.05. Additionally, Disease Ontology (DO) enrichment was conducted on DE-MRGs leveraging both "clusterProfiler" and "DOSE" in R.

#### Candidate diagnostic biomarker screening

Among the three datasets, GSE13507 stands out with a notably larger sample size. Consequently, we opted for GSE13507 as the dataset for constructing our diagnostic model. The extensive sample size offers a wealth of data, enabling the model to comprehensively learn the intricate relationship between features and diseases, thereby mitigating model bias and enhancing the accuracy of disease prediction and diagnosis. In our pursuit of defining vital diagnostic indicators, we engaged two distinct machine-learning methodologies to forecast the disease trajectory. Employing linear regression techniques, LASSO (Least Absolute Shrinkage and Selection Operator) streamlines feature selection and optimizes model regularization, especially in contexts saturated with predictor variables. This balance between data adherence and penalization brought about by LASSO trims away inconsequential variables, preserving only the meaningful predictors. Utilizing the “glmnet” package in R, LASSO regression discerned genes with a pronounced correlation between BC and normal samples. On another front, the Support Vector Machine (SVM) stands as a renowned supervised learning tool in the machine learning realm, predominantly for categorization and regression endeavors^27^. SVM thrives on distinguishing data clusters of varied classes via a hyperplane, ensuring the maximum buffer zone between this demarcation and the nearest, or support, vectors. Introducing the Recursive Feature Elimination (RFE) algorithm was a strategic move to cherry-pick the most representative genes from the accumulated data. Marrying the foundational tenets of SVM with the iterative selection process of RFE, SVM-RFE is geared towards determining the pivotal gene subset within an SVM categorization construct. The synergy fortifies model accuracy, curbs overfitting, and accentuates clarity. Therefore, in our quest to spotlight genes with unmatched discriminatory prowess, SVM-RFE took the lead in feature discernment. Any gene crossovers identified by both computational methods were retained, and the expression metrics of these selected genes underwent further validation using datasets GSE3167 and GSE37816.

#### Single-gene gene set enrichment analysis (GSEA) enrichment analysis

In bioinformatics, GSEA serves as a pivotal tool to deduce the associations and functions of gene clusters in extensive gene expression datasets. The approach offered insights into how variances in gene expression under diverse conditions correlate with certain biological roles, pathways, or phenotypes. Central to GSEA's methodology is the ranking of genes based on their expression fluctuations and subsequently probing if established gene clusters manifest a strong presence in this ordered list. Using the GSEA (V.4.1.0) R package, we evaluated the GSE13507 dataset to explore pathways linked to seven marker genes. By measuring the correlation between these marker genes and other genes in the dataset, we formulated a ranked gene set for testing. Concurrently, we employed the KEGG signalling pathway as a reference set to investigate its prominence within our test set.

#### Evaluation of tissue-infiltrating immune cells

CIBERSORT offered a computational strategy that utilizes gene expression data to determine the proportional distribution of varied cell types within intricate cellular mixtures^28^. It operated by discerning the proportional representation of specific cell types in a given sample, based on the unique expression markers of each cell type. For our study, we harnessed the CIBERSORT deconvolution method to discern the cellular makeup in tissue samples, referencing the established LM22 (leukocyte signature matrix). To ensure robust and consistent outcomes, we set the permutation parameter (perm) to 1000.

### Statistical analyses

To gauge disparities between the two sets, we employed the Student's t-test methodology. Data representation was as the average with an added standard error of the mean (SEM) for clarity. For the purpose of statistical evaluations and crafting visual data displays, we turned to GraphPad Prism 7.0 (GraphPad Software, Inc., La Jolla, CA, USA) alongside R 4.1.0. Adobe Illustrator (AI) CS6 was utilized as the platform of choice to meticulously refine and enhance the graphical illustrations. Difference in data were considered statistically significant if *P* value was < 0.05.

## Results

### Identification of DE-MRGs in BC specimens

**Figure 1:**
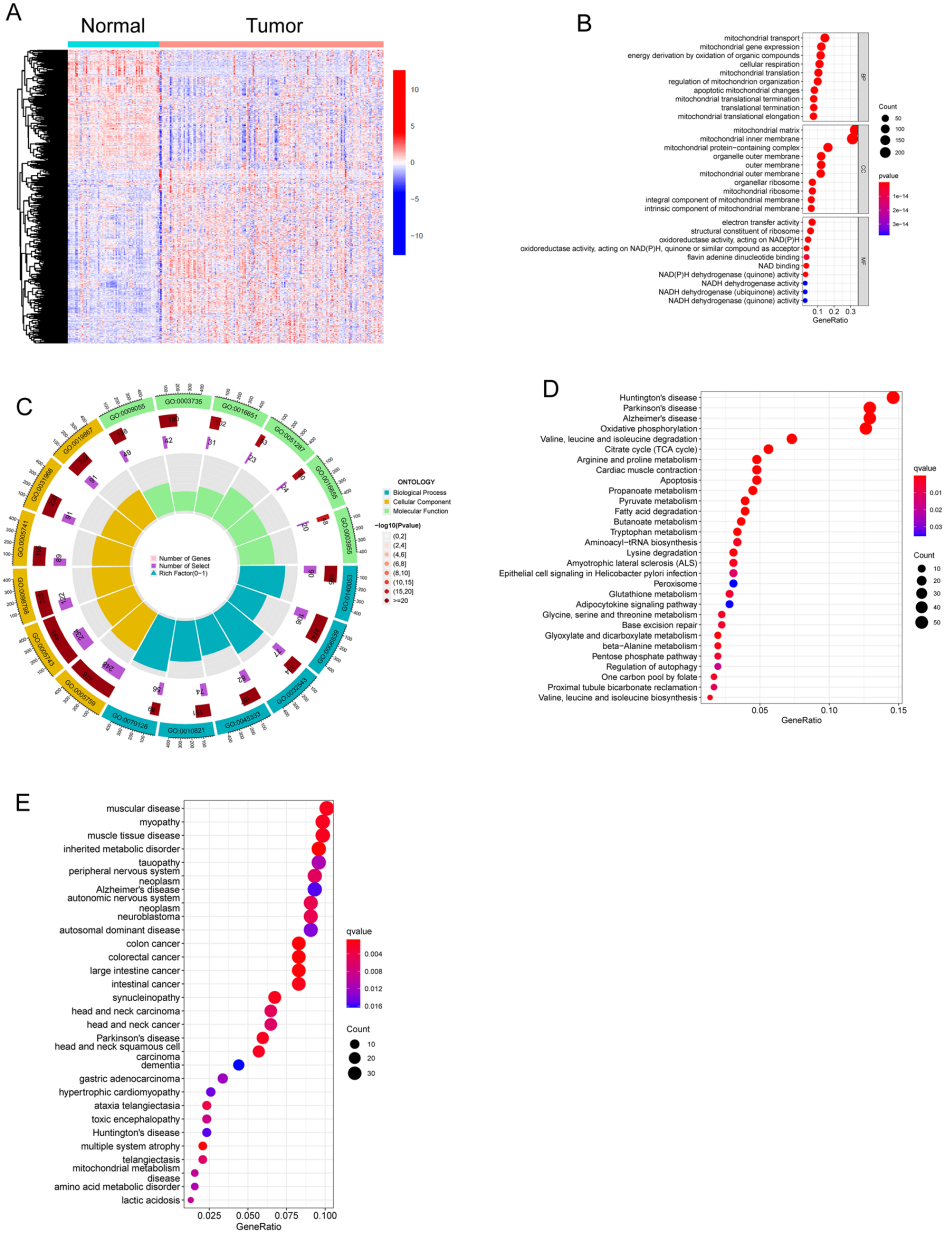
Identification of DE-MRGs in BC patients and Functional Correlation Analysis. (**A**) Violin plots depicted the expression patterns of DE-MRGs between BC specimens and non-tumor specimens. (**B** and **C**) GO enrichment analyses revealed the functional roles of identified DE-MRGs. (**D**) KEGG enrichment analyses. (**E**) DO enrichment analyses.

In our retrospective analysis, we included 165 BC samples and 67 control specimens from the GSE13507 dataset. The evaluation of differential expression of MRGs in these datasets was conducted using the limma tool. From our analysis, a sum of 752 distinct DE-MRGs surfaced. Among these, 439 exhibited notable upregulation patterns, whereas 313 displayed evident downregulation trends, as illustrated in Fig. 1A.

### Functional correlation analysis

In our effort to elucidate the underlying roles of the 752 DE-MRGs in BC, we conducted a comprehensive Functional Correlation Analysis. Key findings from Fig. 1B,C revealed that the DE-MRGs predominantly converged on processes related to pattern specification, regionalization, and embryonic organ formation. They also played roles in cell fate decisions and were found within complexes related to transcription regulation, notably the RNA polymerase II complexes. The involvement extended to both DNA-binding transcription activator and repressor activities, with a specific emphasis on RNA polymerase II. On a broader spectrum, KEGG pathway analysis (Fig. 1D) demonstrated the association of these genes with neurodegenerative disorders like Huntington's, Parkinson's, and Alzheimer's diseases. Additionally, they seemed to influence metabolic pathways such as Oxidative phosphorylation, Valine, leucine and isoleucine breakdown, and the TCA cycle. Delving deeper with DO analysis (Fig. 1E), a significant association emerged with muscular conditions including myopathies and muscle tissue diseases, as well as inherited metabolic anomalies.

### Identification of optimal diagnostic gene biomarkers for BC

**Figure 2:**
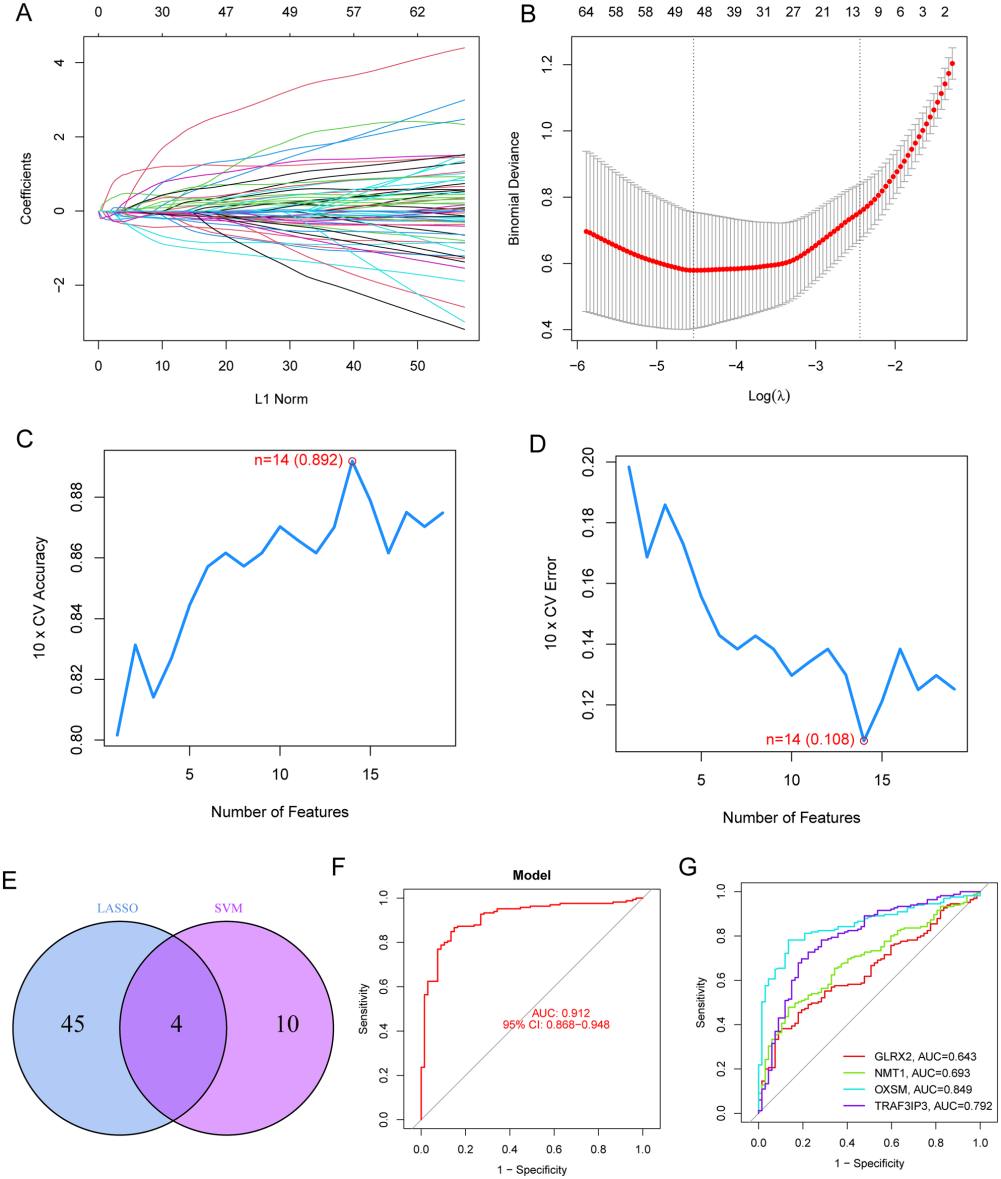
Four DE-MRGs were identified as diagnostic genes for BC. (**A** and **B**) The LASSO logistic regression technique was employed to choose 49 features associated with BC, accompanied by fine-tuning the penalty parameter through a process of tenfold cross-validation. (**C** and **D**) The SVM-RFE algorithm was utilized for sifting through DE-FRGs, aiming to pinpoint the most suitable set of feature genes. Ultimately, a selection of 13 genes emerged as the prime candidates for optimal feature genes. (**E**) The genes identified as markers through the employment of LASSO and SVM-RFE methodologies. (**F**) Employing a logistic regression model to ascertain the AUC for samples from the diseased group. (**G**) ROC curves delineating the performance of the four designated marker genes in distinguishing and classifying specific conditions. These curves visually illustrate the sensitivity and specificity trade-off, offering insights into how effectively the markers can differentiate between different states or groups within the dataset.

**Figure 3:**
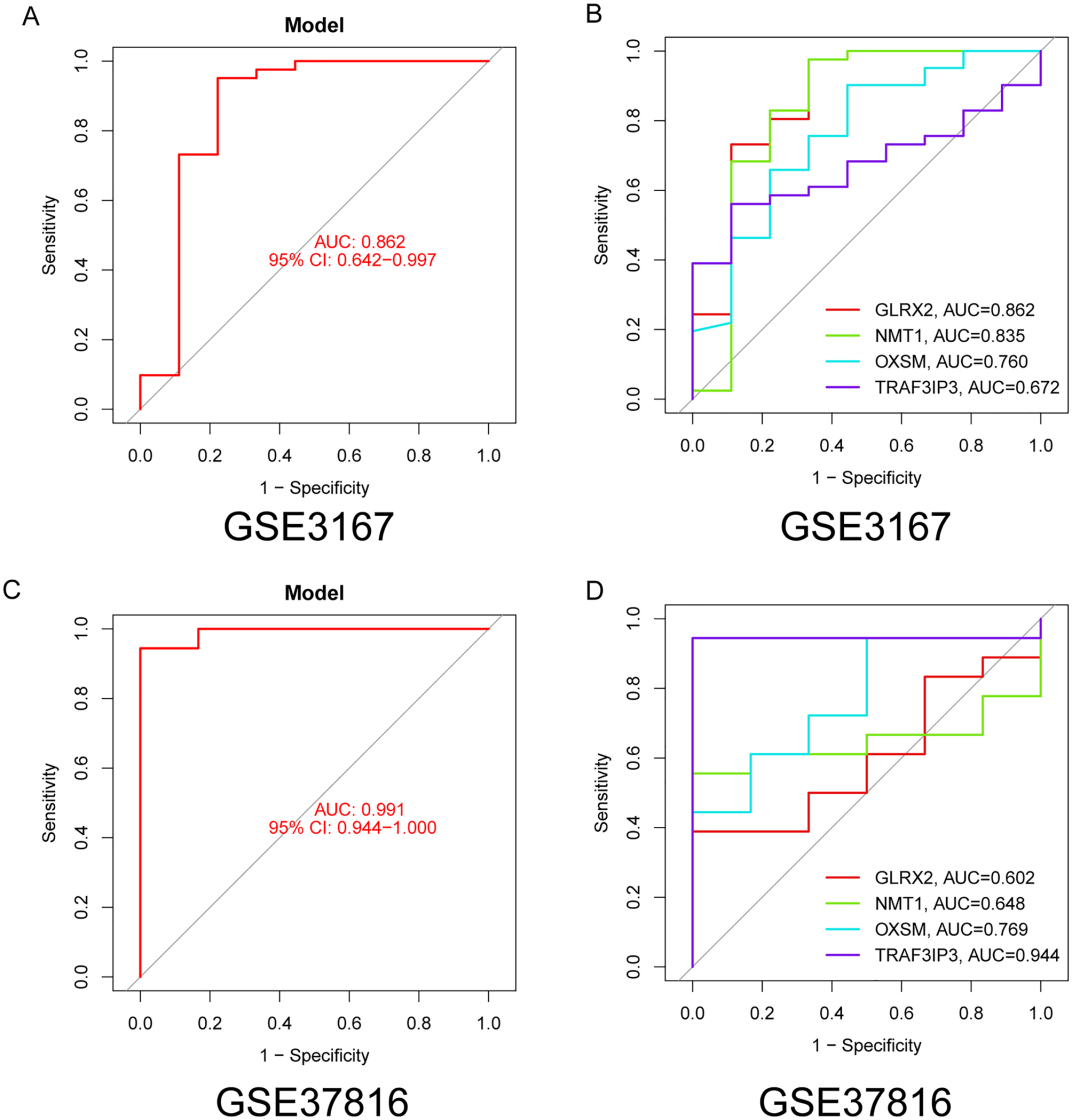
The novel model's diagnostic value was proven using (**A** and **B**) GSE3167 datasets and (**C** and **D**) GSE37816 datasets.

In light of disparities between BC patients and healthy individuals, our study sought to discern the diagnostic capabilities of DE-MRGs. We employed dual machine learning methods, LASSO and SVM-RFE, on the GSE13507 dataset for an effective differentiation between BC and control samples. Utilizing a LASSO logistic regression approach with penalty parameter adjustments through a tenfold cross-validation, we pinpointed 49 significant BC-related features (Fig. 2A,B). Subsequently, the SVM-RFE technique refined our list from the 752 DE-MRGs, zeroing in on an ideal set of 13 genes (Fig. 2C,D). A comparison of outcomes from LASSO and SVM-RFE highlighted five pivotal genes, including TRAF3 Interacting Protein 3(TRAF3IP3), Glutaredoxin 2(GLRX2), 3-oxoacyl-ACP synthase, mitochondrial (OXSM) and N-myristoyltransferase 1(NMT1) for future assessments (Fig. 2E). A logistic regression framework, formulated using the R glm package, centered on these four genes showcased a distinguishing ability between BC and control specimens, recording an AUC of 0.912 (Fig. 2F). To further delineate the diagnostic prowess of individual genes, we charted ROC curves for the seven selected markers. Figure 2G revealed that each gene surpassed an AUC of 0.65. The diagnostic competence of our newly diagnositc model was also corroborated in the GSE3167 dataset (Fig. 3A,B) and GSE37816 (Fig. 3C,D). Cumulatively, the data underscored the enhanced precision and specificity of the logistic regression model in distinguishing BC samples over standalone marker genes.

### The expressing pattern of GLRX2, NMT1, OXSM and TRAF3IP3 in BC patients

**Figure 4:**
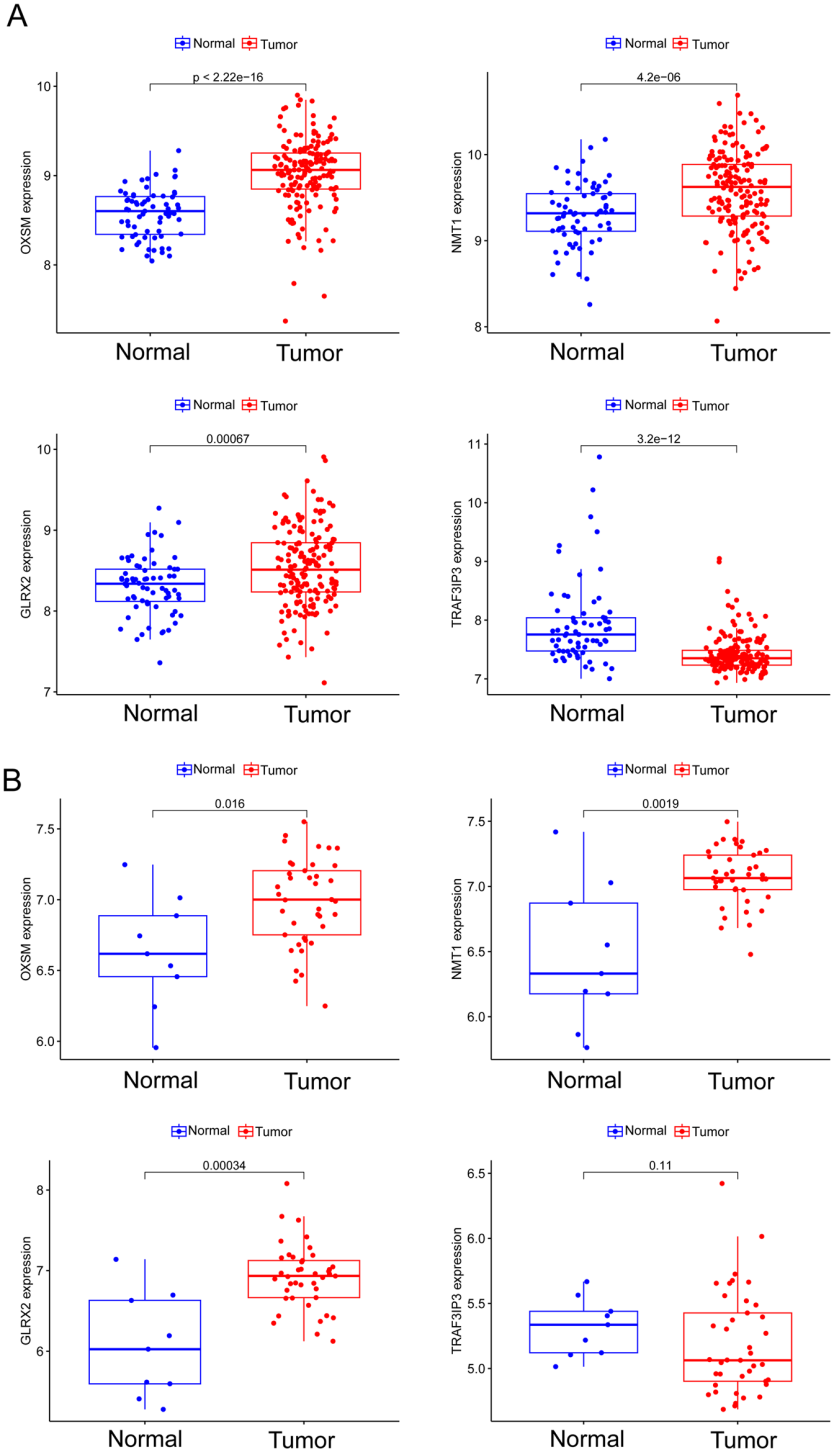
The expressing pattern of GLRX2, NMT1, OXSM and TRAF3IP3 in BC samples and normal samples from (**A**) GSE13507 and (**B**) GSE3167 datasets.

Subsequently, we delved into the expression profiles of the genes GLRX2, NMT1, OXSM, and TRAF3IP3 within BC patients. Based on the data presented in Fig. 4A,B, it was evident that GLRX2, NMT1, and OXSM manifested elevated expression levels in BC samples when contrasted with non-tumor samples. Conversely, TRAF3IP3 displayed a marked reduction in its expression within the BC samples.

### Marker genes were closely linked to a variety of BC-related pathways

**Figure 5:**
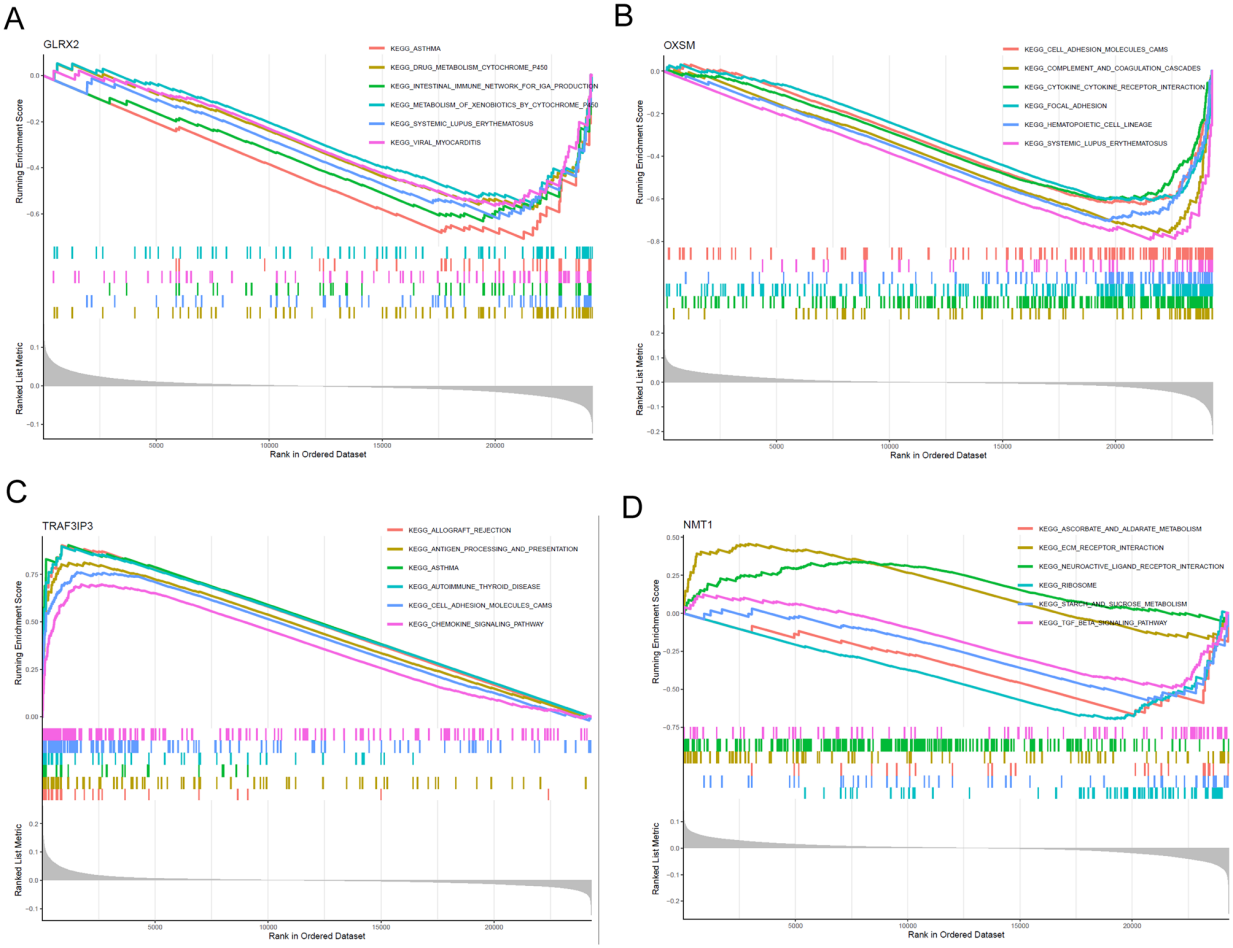
Single-gene GSEA-KEGG pathway analysis in (**A**) GLRX2, (**B**) OXSM, (**C**) TRAF3IP3 and (**D**) NMT1 in BC.

In an endeavor to deeply understand the distinguishing capabilities of the marker genes between disease-afflicted and normal samples, we executed a GSEA-KEGG analysis for each individual gene. Evidently from Fig. 5A, pathways like ASTHMA, DRUG_METABOLISM_CYTOCHROME_P450, and others were more active when GLRX2 expression was subdued. The diminished expression of OXSM seemed to stimulate pathways such as CELL_ADHESION_MOLECULES_CAMS and SYSTEMIC_LUPUS_ERYTHEMATOSUS, as depicted in Fig. 5B. Elevated TRAF3IP3 expression, on the other hand, activated pathways like ALLOGRAFT_REJECTION and CHEMOKINE_SIGNALING_PATHWAY, as observed in Fig. 5C. Lastly, for NMT1, its amplified expression spurred the activation of ECM_RECEPTOR_INTERACTION, while its decreased expression led to the triggering of RIBOSOME and TGF_BETA_SIGNALING_PATHWAY, as shown in Fig. 5D.

### Relationship between the expression of GLRX2, NMT1, OXSM and TRAF3IP3 and immune infiltration

**Figure 6:**
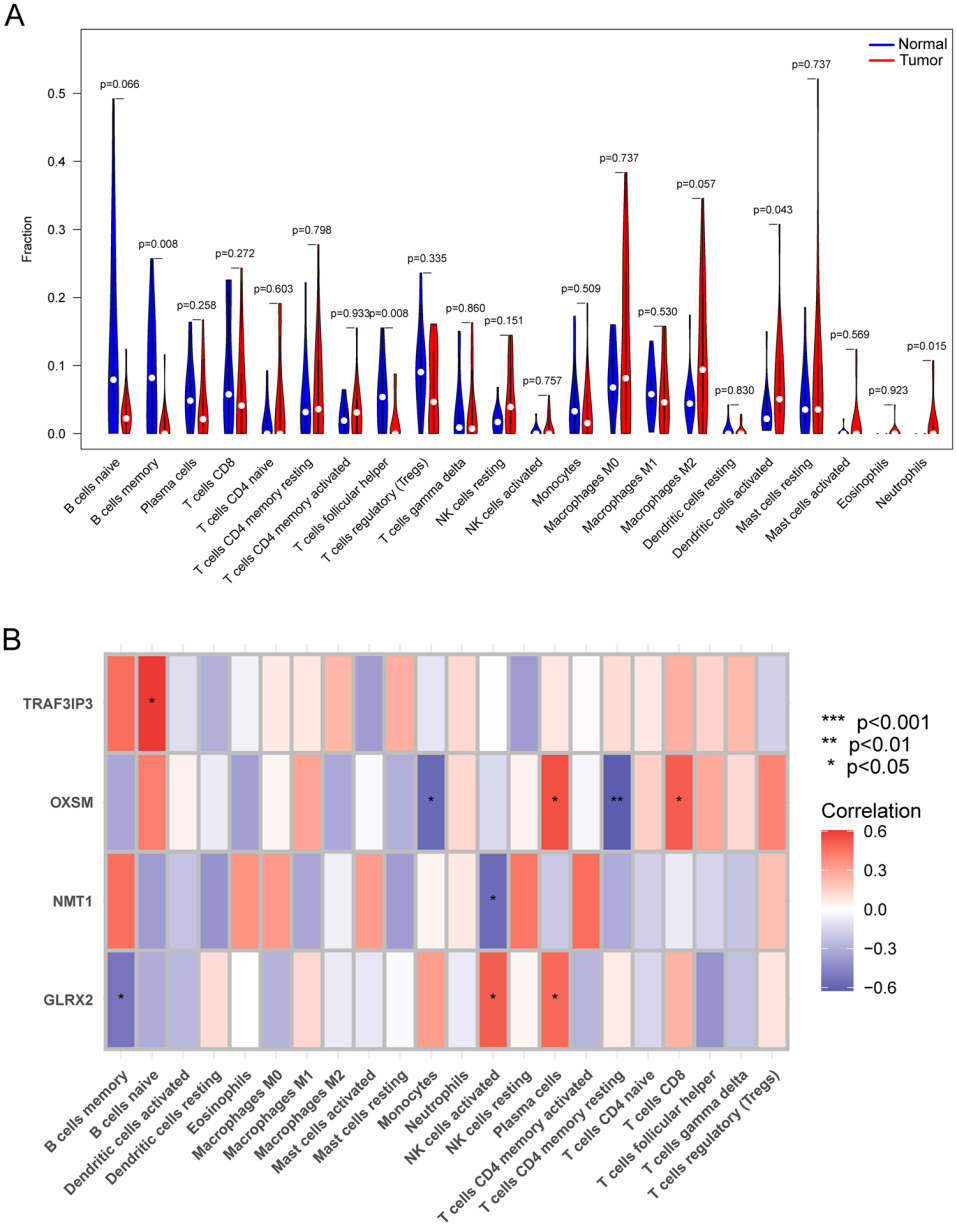
Immune landscape analysis. (**A**)The CIBERSORT algorithm played a pivotal role in uncovering nuanced distinctions within the immune microenvironments of BC patients and normal samples. This computational tool enabled a comprehensive comparison, offering valuable insights into the unique immunological landscapes of these two groups. (**B**) Pearson correlation analysis for GLRX2, OXSM, TRAF3IP3 and NMT1 and immune cells. *p < 0.05, **p < 0.01.

We utilized the CIBERSORT algorithm to delve into the variations present within the immune microenvironment of BC patients when contrasted with standard samples. Illustrated in Fig. 6A, BC samples presented a decline in the representation of both memory B cells, whereas there was a noticeable surge in Dendritic cells that were activated and Neutrophils. Furthermore, the correlation analysis using Pearson's method highlighted a positive linkage between OXSM levels and those of Plasma cells and T cells. Inversely, it unveiled an association in the opposite direction with Monocytes and resting CD4 memory T cells. NMT1 levels showed an inverse correlation with activated NK cells. Notably, GLRX2 levels bore a positive correlation with activated NK cells and Plasma cells. Wrapping up our observations, TRAF3IP3 levels were found to have a direct relationship with naïve B cells (Fig. 6B).

### Knockdown of NMT1 suppressed the proliferation of BC cells

**Figure 7:**
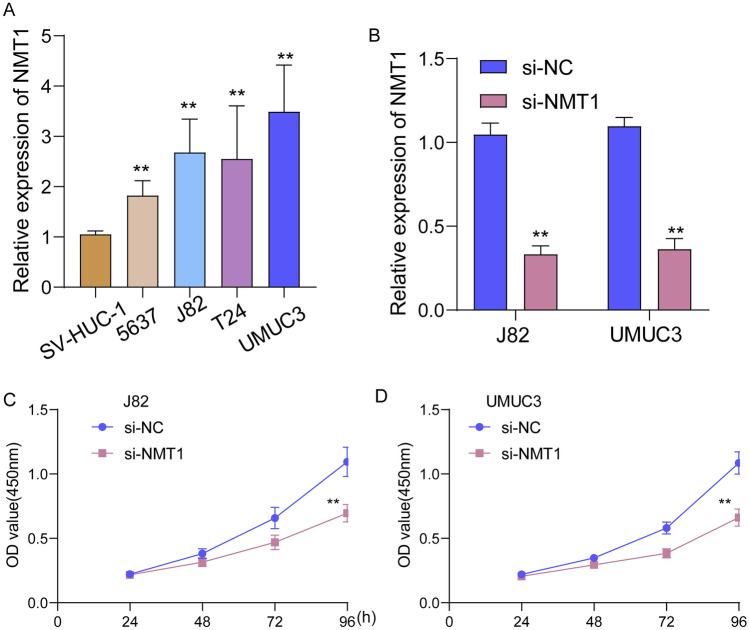
The oncogenic roles of NMT1 in BC progression. (**A**) RT-PCR for the expression of NMT1 in four BC cells and normal cells. (**B**) The expression of NMT1 was decreased in J82 and UMUC3 cells after the transfection of si-NMT1. (**C** and **D**)CCK-8 was applied to examine the effects of NMT1 knockdown on the proliferation of J82 and UMUC3 cells. **p < 0.01.

To ascertain the expression dynamics of NMT1 in BC tissues, we employed RT-PCR techniques. Our analysis showed a pronounced elevation in NMT1 expression across four distinct BC cell lines, as depicted in Fig. 7A. Validating our approach, RT-PCR outcomes verified the efficient si-NMT1 transfection (Fig. 7B). Transitioning our focus, CCK-8 assay results shed light on the impact of NMT1 inhibition. A marked restraint in BC cell growth was evident upon NMT1 silencing, as illustrated in Fig. 7C,D. Our investigation underscored the role of NMT1 as a facilitator in BC's progression.

## Discussion

The diagnosis of BC represents a pivotal medical challenge, encompassing the application of various methods^29^. Presently, diagnostic approaches for BC include clinical symptom analysis, urine testing, imaging examinations, and tissue biopsies^30,31^. Nonetheless, these methods exhibit limitations in terms of early detection, accuracy, and invasiveness. While clinical symptom analysis and urine testing can capture potential BC symptoms and cellular information, their specificity and sensitivity need improvement to mitigate the risk of misdiagnosis or missed diagnosis. Although imaging techniques offer insights into tumor location and size, their efficacy in detecting early lesions remains constrained, often demanding prolonged time and considerable costs. Conversely, tissue biopsies, the "gold standard" for diagnosing BC, entail invasive procedures that cause patient discomfort and carry risks of complications. Furthermore, a reliable non-invasive method for early BC screening is lacking^32,33^. Hence, a pressing need arises to research and develop innovative technologies and methods, such as the integration of machine learning with transcriptome sequencing. This integration holds the promise to enhance the accuracy and early detection rate of BC diagnosis, ultimately offering improved medical services to patients. Overall, while the field of BC diagnosis confronts several challenges, it concurrently provides an opportunity to explore inventive diagnostic strategies and methodologies. Thus, identification of novel sensitive biomarker is very important the clinical prognosis of BC patients.

The critical role of mitochondria within cells goes beyond energy production, encompassing various biological processes, including cell survival, apoptosis, and signal transduction. Consequently, mitochondria may play a central role in tumor development, including BC. Several studies suggest a potential link between mitochondrial dysfunction and BC^34,35^. Tumor tissues from BC patients might exhibit abnormalities in mitochondrial function, including mitochondrial DNA mutations, alterations in mitochondrial membrane potential, and increased oxidative stress. These alterations have the potential to impair mitochondrial energy production and disrupt apoptotic pathways, thereby promoting the survival and proliferation of cancer cells. Moreover, BC progression is intricately connected to changes in metabolic pathways, which may also be associated with mitochondrial dysfunction. Some research suggests that tumor cells tend to favor glycolysis for energy production over oxidative phosphorylation. This shift in metabolic pathways, known as the " Warburg effect," could be influenced by changes in mitochondrial function^6,36^. In this study, we analyzed GSE13507 datasets and identified 752 DE-MRGs in BC patients. Through functional correlation analysis of 752 DE-MRGs, we have revealed their potential roles in the progression of BC. The analysis results indicated that these DE-MRGs were primarily involved in biological processes related to pattern specification, cell fate commitment, and transcription regulator complexes, which are closely associated with cell development and gene regulation. Additionally, KEGG pathway analysis has uncovered associations between these genes and neurodegenerative diseases (such as Huntington's disease, Parkinson's disease, Alzheimer's disease), cellular energy metabolism (oxidative phosphorylation), as well as metabolic pathways (such as valine, leucine, and isoleucine degradation, and the citrate cycle). Furthermore, the DO analysis indicated a correlation between these DE-MRGs and diseases such as muscular disorders, myopathy, muscle tissue diseases, and inherited metabolic disorders. In conclusion, the 752 DE-MRGs may participate in diverse biological processes and pathways during the progression of BC. These processes encompass cell development, gene regulation, energy metabolism, and neurodegenerative diseases. These findings suggested the intricate involvement of these genes in BC development, potentially influencing tumor growth, progression, metabolic anomalies, and associations with other diseases.

Machine learning combined with transcriptomic data offers several advantages in the screening of tumor biomarkers compared to traditional methods^37^. First, machine learning can handle high-dimensional transcriptomic data by extracting essential features to accurately identify gene expression patterns relevant to tumors. Second, machine learning can capture intricate nonlinear relationships and interactions among genes, unveiling molecular mechanisms underlying tumor development, which traditional methods may overlook. Moreover, machine learning enables personalized biomarker selection, tailoring diagnostic and treatment plans based on patients' transcriptomic data, thus enhancing precision^38,39^. In the realm of large-scale data analysis, machine learning's efficient processing capabilities are better equipped to uncover crucial information hidden within extensive datasets, providing timely decision support. Simultaneously, machine learning techniques rapidly generate predictive models, expediting decision-making processes with increased efficiency compared to traditional methods. Furthermore, machine learning can uncover novel biological insights, offering clues to new mechanisms of tumor development and guiding further research and therapeutic strategies^40,41^. Overall, the amalgamation of machine learning and transcriptomic data in tumor biomarker screening offers advantages by delivering more accurate, comprehensive, and personalized information, thereby revolutionizing tumor diagnosis and treatment. In this study, we performed LASSO and SVM-RFE, and identified four critical diagnostic genes, including GLRX2, NMT1, OXSM and TRAF3IP3. Then, we used the above four genes and developed a novel diagnostic model. Its diagnostic value was further confirmed in GSE13507, GSE3167 and GSE37816 datasets. For BC, these findings hold significant clinical implications and potential application value. Firstly, the identification of these four diagnostic genes suggests their potential pivotal role in early detection and confirmation of BC. Secondly, the development of a novel diagnostic model held the promise of providing a more precise and reliable means of diagnosing BC, thus aiding healthcare professionals in better assessing disease progression and treatment strategies. Furthermore, our findings offered substantial support for the investigation of the molecular mechanisms underpinning BC. It has the potential to uncover the latent mechanistic roles of these diagnostic genes in the progression of BC. In summary, this research paved the way for new approaches to early detection and diagnosis of BC, providing valuable insights for the advancement of precision medicine and personalized treatment.

GLRX2 is a protein closely associated with mitochondrial function and redox balance. It belongs to the glutaredoxin family of proteins, whose members exhibit redox activity within cells, aiding in the maintenance of cellular redox states and thereby sustaining normal biological functions^42,43^. GLRX2 is primarily localized within mitochondria, allowing it to play a crucial role in regulating mitochondrial redox balance and other mitochondrial functions. Its structural features enable it to switch between oxidized and reduced forms, participating in redox reactions. As a member of the mitochondrial glutaredoxin family, GLRX2 is involved in ensuring proper protein folding, redox state, and related biological functions within mitochondria, contributing to the maintenance of normal mitochondrial functions, including energy production processes such as ATP synthesis^44,45^. To data, the potential function of GLRX2 in BC was rarely reported. In this study, we found that GLRX2 was highly expressed in BC specimens. The low GLRX2 expression group exhibited an activation trend in several biological processes and diseases, including asthma, drug metabolism (via the cytochrome P450 pathway), IgA production in the intestinal immune network, xenobiotic metabolism (via the cytochrome P450 pathway), systemic lupus erythematosus, and viral myocarditis. These findings suggested a potential association between low GLRX2 expression and the aberrant activation of these biological processes, as well as the development of multiple diseases. However, further research was required to confirm specific mechanisms and interrelationships. These discoveries contributed to a deeper understanding of GLRX2's roles in biology and disease development. In addition, we found that the levels of GLRX2 were positively associated with NK cells activated and Plasma cells. The study found a positive connection between GLRX2 levels and activated NK cells as well as plasma cells, suggesting that GLRX2 might play a role in boosting NK cell activity and contributing to immune responses. Additionally, the link between GLRX2 and plasma cells hinted at its potential involvement in regulating immune reactions and inflammation. These findings could point towards GLRX2 as a potential biomarker for monitoring immune system activity and response. Further research was needed to fully comprehend the mechanisms underlying these associations.

TRAF3IP3 is a gene that encodes a protein which plays a significant role in various cellular functions, including signal transduction, apoptosis (programmed cell death), and inflammation in biological processes^46,47^. AIP1 typically interacts with proteins like TRAF3 (Tumor Necrosis Factor Receptor-Associated Factor 3) and RIP1 (Receptor-Interacting Protein 1), participating in the regulation of multiple signaling pathways. Among these, TRAF3 is a signaling molecule that plays a critical role in immune responses mediated by Toll-like receptors, RIG-I-like receptors, and other receptors. AIP1's interaction with TRAF3 may play an important role in regulating these immune signaling pathways^48,49^. Furthermore, AIP1 is believed to have a significant role in the pathway of apoptosis. Apoptosis is a programmed cell death that cells regulate to maintain the normal development and function of tissues and organs. AIP1 may influence intracellular signal transduction and impact the regulation of apoptotic pathways. In recent years, several studies have reported the potential function of TRAF3IP3 in several types of tumors. For instance, Lin et al. reported that high TRAF3IP3 levels in glioma are linked to poorer survival, possibly due to its role in promoting glioma growth through ERK signaling. TRAF3IP3 might serve as a prognostic biomarker for glioma^50^. However, the function of TRAF3IP3 in BC has not been investigated. In this study, we observed that TRAF3IP3 expression was distinctly decreased in BC specimens suggesting it as a tumor promotor in BC. Moreover, we found that TRAF3IP3 may play a role in regulating immune responses, antigen processing and presentation, cell adhesion, and chemokine signaling. These findings indicated that TRAF3IP3 could have significant functions in modulating immunity and cellular communication during the development of BC.

NMT1 is a gene that encodes a protein. The protein encoded by NMT1 plays a crucial role in cellular processes involving protein modification and signal transmission^51,52^. Belonging to the acyltransferase enzyme family, the protein produced by NMT1 is primarily responsible for attaching myristic acid molecules to amino acid residues of other proteins, a process known as N-myristoylation. This common cellular protein modification, N-myristoylation, affects protein localization, interactions, and function. Specifically, NMT1 catalyzes the N-myristoylation reaction, linking myristic acid molecules to amino acid residues of target proteins. This modification can impact various cellular processes, including signal transduction, apoptosis, and protein–protein interactions^53,54^. NMT1's role in these processes is likely associated with regulating the function, stability, and localization of specific proteins. Previously, several studies have reported that NMT1 served as a tumor promotor in several tumors. For instance, Deng et al. showed that blocking N-myristoyltransferase at the genetic level breast cancer cell proliferation, migration, and invasion were all inhibited by NMT1 through the stress-activated c-Jun N-terminal kinase pathway^55^. In BC, elevated NMT1 expression was found to be inversely correlated with overall survival, indicating that NMT1 overexpression is associated with a poor prognosis. Moreover, increased levels of NMT1 were observed to facilitate cancer progression while simultaneously inhibiting autophagy both in vitro and in vivo^56^. Based on our findings, a comprehensive analysis suggested that NMT1 may have a multifaceted role in BC. Elevated NMT1 expression could be linked to interactions involving the extracellular matrix and neuroactive ligand receptor pathways, implying a potential involvement of NMT1 in tumor cell interactions with the extracellular matrix and neuro-pathways. Conversely, reduced NMT1 expression may relate to metabolic pathways (ascorbate and aldarate metabolism, starch and sucrose metabolism) and the TGF-beta signaling pathway, indicating that NMT1 might influence tumor cell metabolism and growth regulation. In this study, we also found that NMT1 was highly expressed in BC specimens and its knockdown suppressed the proliferation of BC cells, which was consistent with previous findings.

However, there were several limitations in this study. Firstly, the GEO datasets were the primary resources for our clinical data. The majority of its patients are either White, Black, or Latinx. Our results should not be generalized to patients of different races without further investigation. The current research was motivated by the statistical analysis of previously collected data; nevertheless, an optimum threshold must be established before the findings may be applied clinically. Secondly, more experiments are needed to determine the role of these essential diagnostic genes and their protein expression levels in the etiology and development of BC.

## Conclusions

We identified four critical diagnostic genes(GLRX2, NMT1, OXSM and TRAF3IP3) for BC patients. In addition, a novel diagnostic model based on GLRX2, NMT1, OXSM and TRAF3IP3 was developed. Our findings could potentially lead to enhanced accuracy and reliability in diagnosing BC, contributing to more personalized and effective medical interventions for patients in the future. In addition, collaboration with researchers and healthcare institutions on a global scale could expand the applicability of the new diagnostic model and validate its effectiveness across diverse populations.

## Data Availability

The datasets generated during and/or analyzed during the current study are available from the corresponding author upon reasonable request.
